# The concept of the comorbidity model in predicting laparoscopic cholecystectomy results in patients with acute cholecystitis

**DOI:** 10.25122/jml-2022-0237

**Published:** 2022-12

**Authors:** Ihor Yakovych Dzyubanovsky, Yulia Viktorivna Zaporozhets, Nataliia Anatoliivna Melnyk, Svitlana Romanivna Pidruchna, Oleg Ihorovych Dzyubanovsky, Michael Ivanovich Sheremet

**Affiliations:** 1 Department of Surgery, Institute of Postgraduate Education, I. Horbachevsky Ternopil National Medical University, Ternopil, Ukraine; 2 Department of General Hygiene and Ecology, I. Horbachevsky Ternopil National Medical University, Ternopil, Ukraine; 3 Department of Medical Biochemistry, I. Horbachevsky Ternopil National Medical University, Ternopil, Ukraine; 4 Department of L.Ya. Kovalchuk Department of Surgery No.1, Urology, Minimally Invasive Surgery and Neurosurgery, Ternopil, Ukraine; 5 Department of Surgery No.1, Bukovinian State Medical University, Chernivtsi, Ukraine

**Keywords:** acute cholecystitis, laparoscopic cholecystectomy, comorbidity index

## Abstract

In recent years, there has been an unceasing increase in the number of patients with acute cholecystitis, including those with a complicated course of the disease against the background of concomitant pathology. The aim of the study was to establish the level of comorbidity and its influence on the level of postoperative complications in patients with acute cholecystitis who underwent laparoscopic cholecystectomy. We included 457 patients with acute cholecystitis with accompanying pathology, averaging 64.5±9.74 years. Operative intervention was carried out laparoscopically. Patients who scored 4–3 points were considered favorable, and those who scored 0–2 were considered unfavorable. The assessment of comorbidity was carried out using a special index – the Charlson comorbidity index. The majority of patients had a comorbidity index at 2 points (28.0% of all examined patients), indicating the presence of concomitant pathology in the vast majority of patients. We found that the Charleston comorbidity index increased with age, which indicates a higher frequency of concomitant diseases in older patients. A reliable correlation of medium strength was established (R=0.68; p<0.05) between the age and comorbidity indexes. When predicting the mortality of an experimental cohort of patients with acute cholecystitis who underwent laparoscopic cholecystectomy, it can be predicted that the level of the Charlson comorbidity index correlates with the age of patients while the level of postoperative complications increases.

## INTRODUCTION

In recent years, there has been an unceasing increase in the number of patients with acute cholecystitis (AC), including those with a complicated course of the disease against the background of concomitant pathology. The mortality after emergency operations performed for complications of gastrointestinal diseases in the elderly is about 15%, which allows us to consider polymorbidity (comorbidity) as one of the most important medical and social problems of geriatric and surgical practice. Especially in elderly patients, comorbidity leads to a complex summation of symptoms, which reduces their usual diagnostic value for the doctor or potentiates these manifestations and worsens the course of the underlying disease [1–4]. Thus, the impact of comorbid pathology on clinical manifestations, diagnosis, prognosis, and treatment of many diseases is multifaceted and individual. The combination of diseases, age, and medical pathomorphism significantly changes the clinical picture and the course of the main nosology, in our case AC, the nature and severity of complications, worsens the patient's quality of life, limits or complicates the treatment and diagnostic process [5–8].

Comorbidity affects the prognosis of life and increases the probability of mortality. Within polymorbidity, gallstone disease occupies a special place, significantly worsening the general condition of patients: such patients have worse indicators of quality of life and functional activity. Therefore, the problem of treatment of gallstone disease is relevant today [9–13]. In economically developed countries, cholecystectomy remains the most frequently performed operation. With the emergence of minimally invasive surgical technologies in the treatment of gallstone disease, there is a clear pattern of a significant decrease in the number of traditional cholecystectomies, which, it seems, should have a positive effect on the main statistical indicators (postoperative mortality rate, number of complications etc) and, as a result, on the quality of life of operated patients, which is the main objective criterion of the effectiveness and quality of the provided medical care. Complications associated with traditional laparotomy (wound suppuration, eventration, respiratory disorders, formation of ventral hernias etc) occur in 9–12% of patients. According to the recommendations of the World Association of Emergency Surgeons (2020), laparoscopic cholecystectomy, which is performed in an urgent order and is a more "gentle" method of surgical intervention compared to open laparotomy, is the method of choice for AC treatment [14–16].

Despite this, in recent years, there has not been a significant decrease in the number of postoperative complications and mortality or a decrease in the number of emergency operations. Moreover, the frequency of intraoperative complications when using minimally invasive methods of surgical treatment of gallstones, according to the authors, has slightly increased. This is obviously due to the high comorbidity index. In connection with the stated facts, it is relevant to study the frequency of presence and level of comorbidity in patients with AC based on objective criteria for evaluating the effectiveness of surgical intervention and taking into account the index of comorbidity in patients with this pathology.

The aim of the study was to establish the level of comorbidity and its influence on the level of postoperative complications in patients with AC who underwent laparoscopic cholecystectomy.

## MATERIAL AND METHODS

This study included 457 patients with acute cholecystitis with accompanying pathology, with an age range of 20 to 89 years and an average of 64.5±9.74 years (77% women and 23% men), undergoing inpatient treatment in city hospital No. 2, and operated in Ternopil. Clinical and laboratory examinations were performed on all of them, and the diagnosis of AC was verified. Operative intervention was carried out laparoscopically. The Charleston index was determined for all patients: patients with a score of 4–3 were considered favorable, and those with a score of 0–2 were considered unfavorable. The assessment of comorbidity was carried out using a special index – the Charlson comorbidity index (M.E. Charlson, 1987), which is a score (from 0 to 40) of the presence of certain concomitant diseases and is used to predict mortality in the next 10 years [17–20]. When calculating it, the points corresponding to certain concomitant diseases were summed up, and 1 point is added for every 10 years after the age of 40 (40–49 years – 1 point; 50–59 years – 2 points etc). The number of points received makes it possible to forecast mortality, which in the absence of comorbidity is 12%; with 1–2 points – 26%; 3–4 – 52%, and with a sum of more than 5 points – 85%. The use of indexes to assess comorbidity facilitates the assessment of the patient's general status from different sides – the prevalence of diseases, their severity, and the impact on 10-year survival, which should be taken into account when choosing treatment tactics.

### Statistical analysis

Statistical processing was carried out using the SPSS^®^v.21.0 application program package and Excel spreadsheet editor. Data are presented as mean and standard error of the mean (M±m). The reliability of quantitative differences between groups was assessed using the Student's t-test (with normal distribution), or in other cases, the non-parametric Mann-Whitney rank test was used. Differences were considered reliable at a significance level of >95%.

## RESULTS

**Figure 1:**
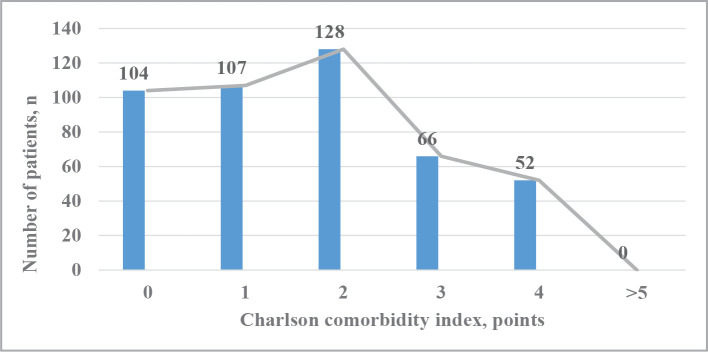
Distribution of patients on AC depending on the sum of Charlson comorbidity index scores.

Evaluating the Charleston comorbidity index, its high indicators were established since the average age of the patients included in the study was 64.5±9.74 years, and the maximum reached 89 years. A similar sample of the age group led to an increase in the comorbidity index and mortality. Thus, the Charleston comorbidity index averaged 1.91±1.9 points, and its indicators ranged from 1 point to 4 points. Figure 1 presents the distribution of patients depending on the sum of Charleston comorbidity index scores. According to the data, the majority of patients had a comorbidity index at 2 points (28.0% of all examined patients), indicating the presence of concomitant pathology in the vast majority of patients.

**Figure 2:**
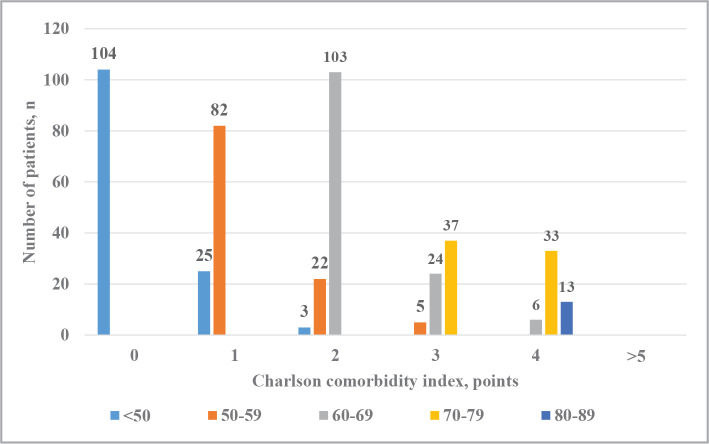
Charlson comorbidity index level of patients with AC depending on their age.

Regarding the age distribution of patients with AC according to the level of the Charleston comorbidity index, we noted that the number of patients with a comorbidity index of 0 points was the largest in the cohort examined under 50 years of age – 22.75% (Figure 2). In addition, the Charlson index was 1 point in 5.47% of the examined patients of this cohort, and 2 points in 0.65% of the patients. Among patients aged 50–59 years, the share of patients with a comorbidity index of 1 point was 17.94%, 2 points – 4.81%, and 3 points – 1.09%. As for the category of patients aged 60–69, 22.53% of patients have a comorbidity index of 2 points, 5.25% – have a comorbidity index of 3 points, and 1.31% –have a level of 4 points. The percentage distribution of patients in the 70–79-year-old cohort was relatively the same: 8.07% had a Charlson comorbidity index of 3 points, and 7.22% had 4 points. Moreover, only a small number of patients in the category of patients 80–89 years old (2.84%) had a Charlson comorbidity index of 4 points.

A significant number of concomitant diseases, on the one hand, complicate the postoperative prognosis and determines the type of surgical intervention, and on the other hand, it has an impact on early and late complications of cholecystectomy. At the same time, the age of patients was the main factor determining comorbidity. We found that the Charleston comorbidity index increased with age, which indicates a higher frequency of concomitant diseases in older patients. A reliable correlation of medium strength was established between the age and comorbidity indexes (R=0.68; p<0.05).

**Figure 3:**
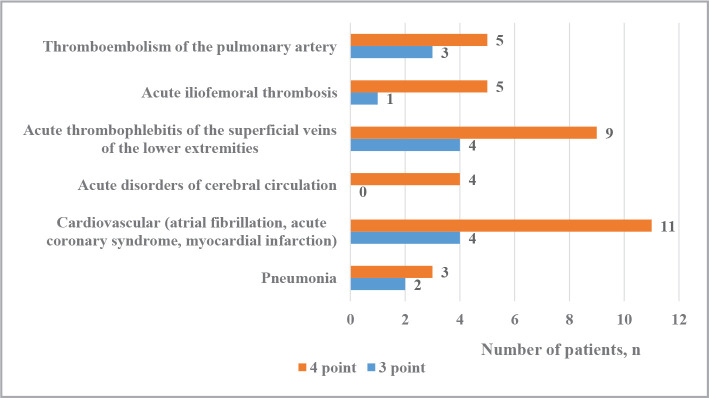
The frequency of postoperative complications depending on the level of the Charlson comorbidity index.

As for postoperative complications after cholecystectomy, the level of the Charleston comorbidity index among the examined patients with AC was within 3–4 points. Among postoperative complications, the most frequently diagnosed cardiovascular pathology (atrial fibrillation, acute coronary syndrome, myocardial infarction) (3.28%) and acute thrombophlebitis of the superficial veins of the lower extremities (2.84%) were diagnosed. Pulmonary artery thromboembolism was diagnosed in 1.75% of patients, and acute iliofemoral thrombosis in 1.31%. Figure 3 shows the distribution of examined patients depending on the frequency of postoperative complications and the level of the Charlson comorbidity index.

As for the index of comorbidity in patients with AC, we can see that it was the highest in patients with cardiovascular complications after cholecystectomy, and its average level was 3.73±0.42 points, 3.73±0.54 points in patients with acute thrombophlebitis of the superficial veins of the lower extremities, 3.62±0.43 points in patients with pulmonary embolism, 3.83±0.53 points in patients with acute iliofemoral thrombosis, and 3.60±0.55 points in patients with pneumonia.

## DISCUSSION

The increase in the number of older people and the combination of two or three, and often even more comorbidities in patients with a surgical profile, in particular in patients with AC, forced doctors to reconsider their views on the treatment tactics of such patients [21, 22]. The fact that various diseases – main, concomitant, or background – lose their mononosological character has become obvious; grouped or not, they seriously change the general status of the patient [23, 24]. Against such an unfavorable background, the diagnostic and treatment tasks of surgeons are significantly complicated. After all, the level of severity of the surgical disease itself and the level of severity of the patient, taking into account the severe concomitant background, can seriously differ and even contradict each other.

In order to understand this issue, statistical data on morbidity have traditionally been used. Initially, these had a general character, where the terminology, the structure of pathological processes, gender, age characteristics, accompanying background etc, were reflected. Later, they were joined by large-scale epidemiological studies of concomitant diseases in various fields of medicine, including surgery, performed using serious statistical calculations. At present, considerable and very diverse material has been accumulated for research into the relationship between the main, background and accompanying pathological processes. As experience accumulated, it turned out that the main and accompanying changes in organs and tissues in various organs during surgical diseases are often connected by a common pathogenesis, previously called the syndrome of mutual burden. It should be noted that this coincided with the first appearance in the press of the concept of "comorbidity", which was initially considered a risk factor affecting the results of operative treatment. Some surgeons [25] already found that an unfavorable background in the form of cardiopulmonary disorders was present in 64.4% of patients, and signs of respiratory insufficiency in older people to one degree or another were found in all patients. In 18% of such patients, liver and kidney failure developed as a result of systemic arterial hypotension and selective changes in the hemodynamics of internal organs, which often occur against the background of chronic diseases (ischemic heart disease, history of myocardial infarction, hypertension) [26].

It is especially demonstrative to model the situation with the mutual influence of diseases on each other using the example of such a very common disease as AC. A lot of statistical and epidemiological data has been accumulated by both surgeons and gastroenterologists, which have long indicated that gallstone disease is closely related to other diseases, the so-called "diseases of civilization" [27]. In such a cohort of patients, the older age group increases especially rapidly; therefore, the condition of such elderly patients due to numerous accompanying processes has long been considered the most difficult. It is also a fact that severe decompensation of concomitant diseases may well bring such patients to a non-operable state [28].

We found that the Charlson comorbidity model provided numerical results in predicting postoperative complications after laparoscopic cholecystectomy in patients with AC [29]. We found cardiovascular and vascular complications often occur in patients after cholecystectomy with a comorbidity level of 3–4 points. Of course, these complications increase the chances of in-patient mortality.

These data suggest that the Charlson index is a valid prognostic tool for risk-adjusting healthcare resource utilization, and researchers should choose more based on their availability and ease of use. Validation of the effectiveness of measuring comorbidity in surgery deserves future study. Further investigation of the Charlson method is an affordable and less costly method of adjusting for mortality risk and postoperative complications. Charlson's comorbidity score for predicting in-hospital mortality and morbidity after laparoscopic surgery should be considered more widely for postoperative mortality studies. Therefore, future studies evaluating and comparing the effectiveness of the comorbidity index to predict long-term treatment outcomes (mortality) would be useful.

## CONCLUSION

When predicting the mortality of an experimental cohort of patients with acute cholecystitis who underwent laparoscopic cholecystectomy, it can be predicted that the level of the Charlson comorbidity index correlates with the age of patients while the level of postoperative complications increases.

## Personal thanks

We want to thank Professor Ihor Yakovych Dzyubanovsky.

## References

[1] Dekker J, Buurman BM, van der Leeden M (2019). Exercise in people with comorbidity or multimorbidity. Health Psychol.

[2] Ukegjini K, Schmied BM (2020). Diagnosis and treatment of acute cholecystitis. Ther Umsch.

[3] Babinets LS, Melnyk NA (2022). Comparative analysis of life quality parameters of patients with a combination of stable coronary artery disease and metabolic syndrome. Rom J Diabetes Nutr Metab Dis.

[4] Sanford DE (2019). An Update on Technical Aspects of Cholecystectomy. Surg Clin North Am.

[5] Dziubanovskyi IY, Pidruchna SR, Melnyk NA (2020). Status of cellular immunity in rats under conditions of acute widespread petitonitis in the setting of diabetes mellitus. Biointerface Res Appl Chem.

[6] Carrasquer A, Peiró ÓM, Sánchez-Giménez R, Lal-Trehan N (2021). Prognostic implications of the Charlson Comorbidity Index and myocardial injury in patients with COVID-19 treated in the emergency department. Emergencias.

[7] Kim SS, Donahue TR (2018). Laparoscopic Cholecystectomy. JAMA.

[8] Strasberg SM (2019). A three-step conceptual roadmap for avoiding bile duct injury in laparoscopic cholecystectomy: an invited perspective review. J Hepatobiliary Pancreat Sci.

[9] Dziubanovskyi IY, Prodan AM, Pidruchna SR, Melnyk NA (2022). Pathogenetic aspects of metabolic syndrome in experimental animals. Wiad Lek.

[10] Babinets LS, Melnyk NA, Shevchenko NO, Migenko BO, Zaets TA (2019). Kallikrein-kinin system disbalance in chronic pancreatis in combination with metabolic syndrome. Wiad Lek.

[11] Babinets LS, Melnyk NA, Kryskiv OI, Korylchuk NI, Nadkevich AL (2020). Metabolic therapy in the complex treatment of chronic pancreatitis with stable coronary artery disease. Wiad Lek.

[12] Dziubanovskyi IY, Pidruchna SR, Prodan AM, Melnyk NA, Palytsya LM (2021). Dynamics of antioxidant status and nitrogen oxide systems in rats with metabolic syndrome after bariatric surgeries. Rom J Diabetes Nutr Metab Dis.

[13] Gomes CA, Junior CS, Di Saverio S, Sartelli M (2017). Acute calculous cholecystitis: Review of current best practices. World J Gastrointest Surg.

[14] Charlson ME, Pompei P, Ales KL, MacKenzie CR (1987). A new method of classifying prognostic comorbidity in longitudinal studies: development and validation. J. Chron. Dis.

[15] Hutor NS, Pidruchna SR, Melnyk NA, Avdeev OV (2020). The role of prooxidant-antioxidant system in the development of alveolitis after teeth extraction. J Int Dent Med Res.

[16] Escartín A, González M, Muriel P, Cuello E (2021). Litiasic acute cholecystitis: application of Tokyo Guidelines in severity grading. Cir Cir.

[17] Lauro A, Cervellera M, D'Andrea V, Casella G (2019). Impact of cardiovascular/diabetic comorbidity on conversion rate during laparoscopic cholecystectomy for acute cholecystitis: a multi-center study on early versus very delayed approach. G Chir.

[18] Tuty Kuswardhani RA, Henrina J, Pranata R, Lim MA (2020). Charlson comorbidity index and a composite of poor outcomes in COVID-19 patients: A systematic review and meta-analysis. Diabetes Metab Syndr.

[19] Varady NH, Gillinov SM, Yeung CM, Rudisill SS, Chen AF (2021). The Charlson and Elixhauser Scores Outperform the American Society of Anesthesiologists Score in Assessing 1-year Mortality Risk After Hip Fracture Surgery. Clin Orthop Relat Res.

[20] Whitmore RG, Stephen JH, Vernick C, Campbell PG (2014). ASA grade and Charlson Comorbidity Index of spinal surgery patients: correlation with complications and societal costs. Spine J.

[21] Gallaher JR, Charles A (2022). Acute Cholecystitis: A Review. JAMA.

[22] Dziubanovskyi IY, Pidruchna SR, Verveha BM, Zhulkevych IV (2021). Morphological characteristics of lungs with experimental peritonitis on the background of diabetes mellitus. Biointerface Res Appl Chem.

[23] Charlson ME, Carrozzino D, Guidi J, Patierno C (2022). Charlson Comorbidity Index: A Critical Review of Clinimetric Properties. Psychother Psychosom.

[24] Pidruchna SR, Benedyct VV, Piatnochka VI, Melnyk NA, Mykhailivna Zakharchuk (2021). Changes of pro-and antioxidant indicators in experimental animals under acute small bowel obstructions. J Med Life.

[25] Oliveira VC, Oliveira P, Correia M, Lima P (2021). Prognostic Value of Charlson Comorbidity Index in Acute Embolic Lower Limb Ischaemia Patients. Ann Vasc Surg.

[26] Lee SO, Yim SK (2018). Management of Acute Cholecystitis. Korean J Gastroenterol.

[27] Halpin V (2014). Acute cholecystitis. BMJ Clin Evid.

[28] Bagla P, Sarria JC, Riall TS (2016). Management of acute cholecystitis. Curr Opin Infect Dis.

[29] Ukegjini K, Schmied BM (2020). Diagnosis and treatment of acute cholecystitis. Ther Umsch.

